# Survey of Slaughtered Pigs for Occurrence of Ochratoxin A and Porcine Nephropathy in Serbia

**DOI:** 10.3390/ijms9112169

**Published:** 2008-11-07

**Authors:** Dragan Milićević, Verica Jurić, Srđan Stefanović, Milijan Jovanović, Saša Janković

**Affiliations:** 1 Institute of Meat Hygiene and Technology, Kaćanskog 13, 11000 Belgrade, Serbia; 2 Department for Animal Sciences, Faculty of Agriculture, University of Novi Sad, Trg Dositeja Obradovića 10, 21000 Novi Sad, Serbia. E-Mail: vjuric@polj.fac.ac.yu; 3 Department of Pathomorphology, Faculty of Veterinary Medicine, University of Belgrade, Bulevar Oslobo enja 18, 11000 Beograd, Serbia. E-Mail: milijan@vet.bg.ac.yu

**Keywords:** Ochratoxin A, pig, tissues, nephropathy

## Abstract

Samples of blood, kidney and liver were randomly selected from slaughtered pigs (n=90) and analyzed for ochratoxin A by HPLC. In addition, in order to obtain information on the occurrence of nephropathy, histological examinations were carried out. Of the 90 liver samples, 26.6% contained OTA in the range of 0.22–14.5 ng/g. The incidence of OTA in serum and kidney were very similar (31%, 33.3%), with a maximum concentration of 220.8 ng/mL, and 52.5 ng/g, respectively. Histopathological examination of kidneys confirmed tubulopathies with edema and cell vacuolization. In addition, hemorrhages and necrosis of proximal kidney tubules’ cells were found.

## 1. Introduction

Ochratoxin A (OTA) is a nephrotoxic mycotoxin produced by several species in the *Aspergillus* and *Penicillium* genera. These species occur worldwide, as a contaminant of agricultural commodities, especially cereals [1], but also in a variety of other food commodities [2, 3] as a result of poor storage of said commodities or poor agricultural practice during drying procedures [4]. OTA is nephrotoxic to all animal species and causes mycotoxicosis in animals, particularly in swine [5, 6] and this mycotoxin plays a special role in the genesis of mycotoxic porcine nephropathy (MPN), a common disease in Scandinavia [5]. OTA has been considered as possible cause of the human disease known as Balkan Endemic Nephropathy (BEN) [7]. The toxicological profile of OTA includes teratogenesis, nephrotoxicity and immunotoxicity [8]. Moreover, OTA was classified as being carcinogenic in animals [9–11]. Special attention has been paid to OTA since 1993, when the International Agency for Research on Cancer classified this toxin as a possible human carcinogen (group 2B) [12–14]. Under certain circumstances the levels of OTA in pig blood are of great concern. Hult *et al.* (1980) demonstrated that the level of OTA in blood is very expressive of the general exposure of the individuals to this mycotoxin and would be a useful tool in diagnosing ochratoxicosis. The high affinity of OTA to proteins, particularly to serum albumin, allows its accumulation in the organs of animals [16]. In fact, animal-derived products and tissues for human consumption may well present OTA residues even if the animal has been nourished with feeds contaminated with low levels of OTA. In many studies, OTA has been detected in pig’s blood, kidney, liver muscle and adipose tissue with rather high levels found in animals suffering from porcine nephropathy [6, 17–22], especially in countries of Balkan Peninsula [23–27]. A nephropathy in pigs with characteristic macroscopic changes of the type “*mottled or pale enlarged kidneys”* has been frequently identified at meat inspection in Serbia, corresponding well with the data found in other countries of the Balkan Peninsula [24].

The purpose of this work was to monitor the presence of OTA in edible tissues of normally slaughtered Serbian swine, and also to evaluate data for the relation between the content of ochratoxin A and pathomorphological changes in kidney.

## 2. Results and Discussion

### 2.1. Occurrence, concentration and regional distribution of Ochratoxin A in tissues

The occurrence and mean concentrations of ochratoxin A in swine serum, kidney and liver are summarized in Tables 1 and 2.

#### 2.1.1. Ochratoxin A in Serum

OTA contamination assessment showed that 28 (31%) of the analyzed serum samples (n *=* 90) were contaminated in a very wide range from 0.22 to 220.8 ng/mL (mean levels 3.70 ± 23.59 ng/mL). The incidences of OTA and mean level of contamination in the three regions where samples were collected are very different (Table 1), varying between 16.6% (mean-0.19 ng/g, Vladimirci) to 43.3% (mean-2.33 ng/g, Senta). The highest OTA level 220.8 ng/mL (mean-8.58 ng/mL), with the highest coefficient of variation (4.69) was found in the samples originate from the Bogatić region. Analysing the range of OTA levels in fourteen samples (15.5%), levels of ochratoxin A ranged from 0.1 to 1 ng/mL. The concentrations in a further eight samples (8.8%) fell between 1–5 ng/mL while the rest of the samples (6.6%) had concentrations greater than 5 ng/mL. The incidences of higher concentrations of ochratoxin A (> 5 ng/mL) in serum was similar to those in kidney (6.6 and 5.5% respectively), while ochratoxin A was greater than 5 ng/g in only two (2.2%) liver samples (Table 2).

#### 2.1.2. Ochratoxin A in Kidney

In contrast, our results showed that the frequency of contamination of OTA was higher in the kidney than in the serum and liver (Table 1). The incidence of ochratoxin A among the three regions where samples were collected varied between 26.6% (Vladimirci) to 36.6% (Senta and Bogatić) with a mean contamination frequency of 33.3%. In regard to regional distribution of OTA, the average OTA concentration in positive samples varied between 0.42 ng/g (Vladimirci) and 2.2 ng/g (Bogatić) where there is the highest concentration of OTA 52.5 ng/g. The majority of samples (16.6%) contained OTA at low levels (0.01-1 ng/g). The concentrations in a ten samples (11.1%) ranged between 1–5 ng/g, while ochratoxin A in five (5.5%) samples was greater than 5 ng/g (Table 2). In 2.2% samples of kidneys, OTA levels was considerably higher and greatly exceed the permissible levels of this toxins established in Serbia and included those proposed (10 ng/g) by the SCF [33], and JECFA [34].

#### 2.1.3. Ochratoxin A in Liver

In the present study, OTA was detected in 24 (26.6%) out of 90 liver samples with a much lower mean value (0.63 ng/g) than in kidney (1.26 ng/g) (Table 1). The majority of samples (15.5%) contained OTA between 1–5 ng/g, while ochratoxin A in only two (2.2%) samples of liver was greater than 5 ng/g (Table 2). In regard to the regional distribution of OTA, the occurrence of OTA among the regions where samples were collected are different and varied between 13.3% (Senta) to 36.6% (Vladimirci), but the mean level of contamination were very similar (0.48–0.84 ng/g). The highest OTA level 14.5 ng/g (mean OTA 0.84 ng/g), with the highest coefficient of variation (3.51) was found in the samples from Senta.

Comparison with other published data for the occurrence of OTA and contamination level was generally not different from other European countries such as Sweden, Poland, and Germany, or in areas of Balkan Peninsula and Canada (Table 3).

The present work indicates that regional variations and seasonal differences were observed. Geographical origin and season were recognized as the main factor influencing the OTA content of tissues when samples of the three different regions were compared. During period December-February the mean content of OTA in serum samples in the investigated regions was significantly different (p *<* 0.05), while the mean content of OTA in liver samples was highly significantly different (p *<* 0.01). The regional differences and seasonal variations might thus partially explain the concentration differences in the corresponding formulas, as could differences in the storage condition of feedstuffs. In addition, fluctuations in mould growth and contamination level of cereals may result in seasonal variations in dietary exposure to OTA. The highest mean level of OTA found in Bogatić region, could signal a possible relationship between this region and the Balkan Endemic Nephropathy.

With regards to a consumer safety point of view, the literature contains several studies investigating the ration between the levels of OTA in pig kidneys and pig meat [20, 25, 35, 36, 39]. A contamination level for the entire carcass at 25 ng/g pig kidney should secure that the level in meat do not exceed 10 ng/g, based on the estimation that the OTA level in pig meat is approximately 40% of the level in pig kidney. The ration “content in meat/content in kidney” varied between 10 and 90%, and can depend on many factors, e.g. the content of OTA in feed, feeding period, feeding in relation to time of slaughtering. The results from these survey indicated that there was a low correlation between the OTA level in serum and liver as well as in the OTA level in kidney and liver (r = 0.319 and r = 0.341, respectively) while the strongest correlation was found between the OTA level in serum and in kidney (r = 0.973). A similar correlation was found by [39]. The effects of OTA appeared to be longer-lasting than those of other mycotoxins, and possessed cumulative feature. Comparison the data obtained in this trial with other recently published data for the occurrence of OTA in pig edible tissues shows that the found levels are comparable with levels in other European countries [24, 39, 40]. However, it should be remembered when comparing data that factors such as climate conditions during harvest, practices for grain/feed storage etc have influence on the ochratoxin A level in edible tissues. The data obtained in this trial show it should be raises some concern for the livestock industry.

### 2.2. Pathomorphology examination

#### 2.2.1. Gross pathology

**Figure 1.**
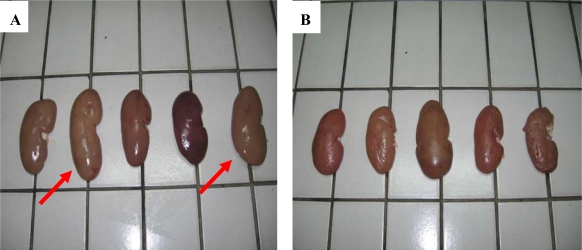
External surface of a case of *“mottled enlarged kidneys”* (A) (second and fifth kidney dorsal aspect), and (B) normal sized kidneys, except for the third.

In all 90 pigs were slaughtered during the study period. Kidneys which were submitted to the laboratory were pale, swollen and enlarged and change in color from the normal mahagony to tan, as follows: 43 (47.7%) had “mottled or pale kidneys”, while 27 (30%) were enlarged (Figure 1) and 11 (12.2%) was smaller than normal. The only other macroscopic lesion in few cases was a small grey-white foci on the kidney surface. No obvious difference was observed between the right and left kidney. No significance changes were seen in other organs.

#### 2.2.2. Histological examination

**Figure 2.**
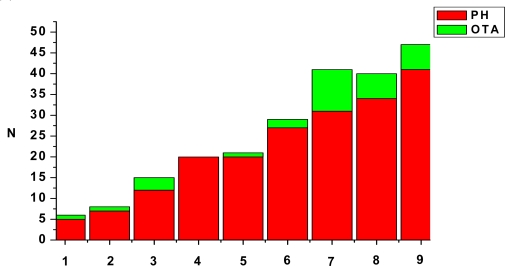
Summary of histological findings (PH) of renal tissues and incidence of OTA in kidney from slaughtered pigs (n=90). Necrosis of proximal tubules’ cells (1), hypercellularity of vascular loop (2), vascular changes (3), exudat in Bowman’s space (4), focal interstitial nephritis (5), dystrophy of proximal tubules’ cells (6), swelling of proximal tubules’ cells (7), renal hemorrhages (8), fatty changes of proximal tubules’ cells (9).

**Figure 3.**
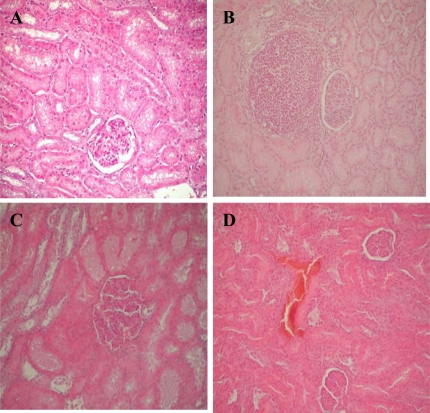
Dystrophy and vacuolar degeneration in the epithelium of proximal tubules’ cells (A), focal interstitial fibrosis (B), necrosis of proximal tubules’ cells (C) and hemorrhages in cortex (D).

Histological examination showed two types of changes: degenerative – affecting epithelial cells in some proximal tubules of pigs, and proliferative changes in the interstitium (Figure 2). The major renal histopathological changes were mainly in the epithelium of proximal tubules. Dystrophy (moderate to marked degenerative changes, Figure 3A), swelling, vacuolization and fatty changes, were the main changes in the tubular epithelial cells. The majority of glomeruli exhibited mild or moderate exudates in Bowman’s capsular spaces as well as hypercellularity of vascular loops. In addition vascular changes expresed as a hyperaemia of blood vessels, moderate to marked hemorrhages of some renal cortical regions occurred occasionally (Figure 3D). In the interstitium of some renal cortical regions, there was limited proliferation of connective tissue (Figure 3B) and focal infiltration of mononuclear inflammatory cells which was sometimes accompanied by small granuloms. These results have also been reported in other papers [24, 42–46].

OTA analysis of the kidneys samples where degenerative changes of moderate to marked cloudy swelling were seen revealed the incidence of OTA in 32.2% samples at concentrations levels up to 52.5 ng/g (Figure 2). Additionally, vascular changes as well as fatty changes were observed in six kidneys of pigs were ochratoxin A detected up to 6.5 ng/g, while focal interstitial fibrosis and necrosis of proximal tubules’ cells were only seen in one kidneys of pigs were ochratoxin A detected up to 52.5 ng/g. In comparison, the lesions produced by higher OTA levels were more severe and widespread, including degeneration, atrophy, glomerular swelling and sclerosis and interstitial nephritis (Figure 2).

The kidney is the main target of OTA, although it has been shown that possible targets of OTA are the liver, the immune system, and brain cells [47–49]. This high susceptibility of the kidney is, at least in part, the result of OTA-toxicokinetics. Renal blood flow per tissue weight is extremely high, resulting in the delivery of relative large amounts of OTA as compared to other organs. Furthermore, free OTA is secreted in the proximal tubule and subsequently reabsorbed, mainly in the proximal straight tubule, the thick ascending limb of the loop of Henle and the collecting duct [50–56]. Mechanisms involved in reabsorption are, e.g., H+-dipeptide-cotransporter(s) and non-ionic diffusion [52, 55, 56]. These toxicokinetic features result in an accumulation of OTA in renal tissue, where the highest concentrations have been detected in the papilla and the inner medulla [57]. The inhibition of protein synthesis and the damaged energy production in the mitochondria could be considered as the most important factors for degenerative changes in the epithelial cells of proximal tubules where ochratoxin A was detected [58]. While agreeing that the most important toxicological target of OTA in the pig is the kidney, the principal descriptions of the pathology of MPN vary considerably with respect to some other details, and according to the dosing regime and the duration of OTA exposure. Enlarged kidneys are indicative of renal inflammation and proliferative lesions following chronic exposure to OTA [24, 46]. In the present study no association was found between OTA contamination and kidney weight. Twelve (28%) out of 43 “*mottled or pale kidneys”* samples were found to contain OTA, whereas that in the *enlarged kidney* was 44.4% in the range 0.17–52.5 ng/g. However, the average concentrations of OTA in some feeds for pigs in Serbia (up to 0.25 mg/kg) were substantially lower than the 1–2 ppm required to reproduce the classical MPN reported in Denmark. Also, pigs may well contain ochratoxin A in high amounts without any macroscopic chances of the kidneys, as it may take several weeks on ochratoxin A contaminated feeding before porcine nephropathy develops.

Many studies on the role of environmental and host factors have been carried out to elucidate the aetiology of both diseases (BEN and MPN). Such factors include heavy metals [59–62] and other minerals [63], bacteria, leptospira, viruses [64, 65], fungal toxins and, recently, Pliocene lignite’s [66], but much stronger evidence support the hypothesis that BEN and MPN have mycotoxic aetiology. It seems, therefore, that MPN observed in Serbia may have a multitoxic aetiology, because it cannot be explained by the concentration of OTA alone. The lack of a strong correlation among histopathological changes and incidence of OTA in kidney (33.3% kidneys samples were positive, at levels ranging up to 52.5 ng/g) found in our trial might thus explain the result of OTA-toxicokinetics as well as possible synergism between OTA and other nephrotoxic mycotoxins or compounds which enhance the toxicity of OTA. Such synergism between OTA and other mycotoxins under field conditions may be responsible for the MPN in Serbia, associated with relatively low mean contamination by OTA in feed. The production of multiple toxins by one or several fungi [67], as is sometimes the natural situation, presents a problem that has not been sufficiently investigated. Considering the similarities with the frequency and levels of OTA reported in other countries, it seems that OTA in Serbian slaughtered pigs represent a particular situation.

The timely diagnosis of disease during the meat inspection at slaughterhouses is very important. The Serbian control system for content of OTA in pig kidney is not yet established, and can be regarded as a lack from a consumer safety point of view. However, the actual concentration in pork is generally very low and hence for the consumer the contribution to the total intake of ochratoxin A from pig products is very small compared with other sources [68]. The values are far below the Acceptable Daily Intake (*ADI*) of these toxins [33, 34], but high amounts of very hazardous and relatively heat stable OTA may enter in the food chain without any control system. With the present safety evaluation of ochratoxin A [33, 34], a residue limit of 10 ng/g in pork would not be considered satisfactory from a consumer safety point of view.

The prevalence of mycotoxin-induced nephropathy is still unknown, and epidemiological studies must be carried out on a large scale in livestock population where ochratoxin A levels in feed are high, as they are also relevant for the understanding of human nephropathies.

## 3. Experimental Section

### 3.1. Reagents

OTA (benzene free) were purchased from Sigma-Aldrich Chemie GmbH. Stock concentrated solution was prepared in toluene-acetic acid (99:1 v/v) at final concentration of 1 mg/mL and stored at -20 °C, protected from light. The OTA working solution was prepared by diluting the stock solution with toluene-acetic-acid (99:1 v/v) to ∼10μg/mL. The actual concentration of OTA was calculated using UV spectrophotometer set at 333 nm (ɛ 5,550). After suitable dilutions in water-methanol-acetic acid (50:49:1 v/v/v), the working solution was used to prepare the external calibration curve. A working standard OTA for HPLC was prepared daily just before starting the injection of a series of samples. Other reagents were HPLC grade. All other chemicals were reagent grade or chemically pure.

### 3.2. Sample collection

During six month period (September 2006/February 2007), samples of blood, kidney and liver from each animal were collected from healthy slaughtered pigs (n = 90) originating from three different regions of Serbia where there is a significant swine industry. Slaughtered pigs were randomly sampled in the slaughterhouse during meat inspection. Serum samples were collected from each studied farm and liver and kidneys of corresponding animals. About 50 mL blood/pig was sampled when slaughtered pigs were bled by jugular puncture. Blood samples remained at room temperature for 24 h to allow clotting to occur, and were then centrifuged at 3,000 × *g* for 20 min. Serum was decanted and stored at -20 °C prior to analysis. About 100 g of liver and whole kidney were sampled from each pig. After cutting pieces of kidney for histological examination, the rest of sample was homogenized and stored at -20 °C before analysis. No preservatives were added. For microscopic examination kidney samples were fixed in 10% neutral buffered formalin. and absolute alcohol for 5 to 7 days, processed by routine methods, sectioned at 5–8 μm, and stained with haematoxylin and eosin (HE) for light microscopy.

### 3.3. Extraction and clean-up for ochratoxin analyses

#### 3.3.1. Extraction and clean-up for ochratoxin analyses from serum

Serum (0.8 mL) was extracted according to Curtui and Gareis [25] with 15% trichloroacetic acid (0.2 mL) and dichloromethane (1 mL) by vigorous vortexing for 30 s in a 2 mL safe-lock polypropylene conical-bottom centrifuge tube. The mixture was allowed to stand for 24 h at room temperature, and then centrifuged at 14,000 × g for 10 min. The lower dichloromethane phase was carefully withdrawn by a Pasteur pipette and transferred to a 1.5 mL safe-lock polypropylene conical-bottom centrifuge tube. The acidic phase and the compact precipitate layer formed between the two phases were re-extracted with dichloromethane (0.5 mL) for 30 s on a vortex mixer and then centrifuged for 5 min at 14,000 × g. The pooled dichloromethane extract was evaporated to dryness at 40 °C under a gentle nitrogen flow. The remaining residue was dissolved in methanol (80 mL) and transferred to a 300 μL HPLC vial. The limit of detection (signal/noise: 3/1) was estimated at 0.1 ng OTA/mL, and recoveries was 95%.

#### 3.3.2. Extraction and clean-up for ochratoxin analyses from kidney and liver

Kidney and liver analyses were performed by the method of Matrella *et al.* [69], which briefly includes a double extraction with acidic ethyl acetate. The organic phase was removed and extracted with 0.5M NaHCO_3_, pH 8.4. The aqueous extract was acidified to pH-2.5 with 7M H_3_PO_4_ OTA was finally back extracted into ethyl acetate (3 mL). The organic phase was evaporated to dryness under N_2_ steam, reconstituted in 150 μL mobile phase and a 20 μL aliquot injected. The detection limit for OTA in organs was 0.01 ng/g with a 61% (C.V. =14.5%) mean recovery from artificially contaminated samples at 3 ng/g (n = 3).

#### 3.3.3. Chromatographic conditions (HPLC)

An aliquot of 20 μL for serum samples and 50 μL for kidneys and liver samples were injected onto a Waters Symmetry Shield RP (Reversed phase) 18, high pressure liquid chromatography column (length and inner diameter 150×4, 6 mm, particle size 5 μm) on a Waters Alliance HPLC system. The column was eluted with 4% acetic acid and acetonitrile (32:68 v/v) at 25 °C and a flow rate of 1 mL/min. Measurements were performed by fluorescence detection at wavelengths of 334 nm (excitation) and 460 nm (emission) gains 10. A volume of 10 μL was injected for the standards and 20 μL for the samples. For more accuracy, 40 μL was re-injected in the case of the samples with an amount of OTA near the detection limit.

#### 3.3.4. Confirmation of Ochratoxin A by liquid chromatography tandem mass spectrometric method (LC-MS/MS)

Analyses of serum, kidneys and liver sample were performed by the methods described above [19, 52]. Sample extracts were evaporated to dryness under a gentle stream of nitrogen and stored at -18 °C prior to analysis.

#### 3.3.5. Liquid chromatography tandem mass spectrometric conditions

Mass spectrometry was performed by Micromass Quattro II triple quadrupole mass spectrometer and MassLynx software for control and data processing. Electro spray ionization in the positive mode was used. The electro spray capillary was set at 3.2 kV and the cone at 30 V. The ion source temperature was set at 115 °C and the flow rates for nitrogen bath and spray were 700 l/h and 55 l/h, respectively. Data were acquired in the multiple reaction monitoring (MRM) modes. The collision energy was 18 eV. The transitions reactions monitored by LC-MS/MS were *m/z* 404*→*239 and *m/z* 404*→*358 daughter ion of the ochratoxin A.

### 3.4. Statistical analysis

Descriptive statistics of the data set were performed with a standard programmed and included arithmetic mean, standard deviation, coefficient of variation, minimum, maximum. Statistical differences in the mean levels of OTA contamination across the three groups of positive samples were determined by one-way ANOVA (p<0.05). Significance was set at p*<*0.05.

## Figures and Tables

**Table 1.** Incidence of ochratoxin A in tissues of slaughtered pigs.

Region	N	Serum (ng/mL)			
		n (%)	*X̄* ±Sd	C.V.	Range
Vladimirci	30	5 (16.5)	0.1±90.57	2.96	0.33–2.56
Senta	30	13 (43.3)	2.33±6.91	2.96	0.24–35.7
Bogatić	30	10 (33.3)	8.58±40.25	4.69	0.22–221
**Total**	**90**	**28 (31.1)**	**3.70±23.6**	**6.37**	**0.22–221**
**Kidneys (ng/g)**					
Vladimirci	30	8 (26.6)	0.42*±*1.2	2.96	0.18–6.5
Senta	30	11 (36.6)	1.14*±*3.3	2.89	0.17–17
Bogatić	30	11 (36.6)	2.2±9.54	4.33	0.26–52.5
**Total**	**90**	**30 (33.3)**	**1.26±5.85**	**4.64**	**0.17–52.5**
**Liver (ng/g)**					
Vladimirci	30	11 (36.6)	0.48*±*0.75	1.55	0.32–2.2
Senta	30	4 (13.3)	0.84±2.95	3.51	0.56–14.5
Bogatić	30	9 (30)	0.56±1.17	2.09	0.22–5.46
**Total**	**90**	**24 (26.6)**	**0.63*±*1.87**	**2.96**	**0.22–14.5**

N-total number of analyzed samples, n-number of positive samples, *X̄* -arithmetic mean (conc. below LOD are regarded as zero), C.V.-coeff. of variation.

**Table 2.** Distribution of ochratoxin A in tissues of slaughtered pigs in the regions where samples were collected.

Region	N	Number of samples in the range			
		Serum			
		< LOD	0.1**^b^**-1	1–5	>5
Vladimirci	30	25	3	2	0
Senta	30	17	6	4	3
Bogatić	30	20	5	2	3
**Total**	**90**	**62**	**14**	**8**	**6**
		**Kidneys**			
		< LOD	0.01**^b^**–1	1–5	>5
Vladimirci	30	22	5	2	1
Senta	30	19	5	4	2
Bogatić	30	19	5	4	1
**Total**	**90**	**60**	**15**	**10**	**5**
		**Liver**			
		< LOD	0.01**^b^**–1	1–5	>5
Vladimirci	30	19	5	6	0
Senta	30	26	1	2	1
Bogatić	30	21	2	6	1
**Total**	**90**	**66**	**8**	**14**	**2**

N-total number of analyzed samples, nd-not detectable, ^b^LOD -limit of detection (see Experimental Section).

**Table 3.** Incidence of ochratoxin A in tissues of slaughtered pigs in different countries.

Samples	Country	Incidence %	Mean ppb	Range ppb	References
Serum			6.7		[28]
	Sweden	18			
			9.4		[29]
	Poland	38			[30]
	Germany	48.7	5.8		[3]
	France	2		up to 6	[40]
	Romania	98	2.43	up to 13.4	[25]
	Bulgaria	100	60.9		[24]
	Canada	36	14		[32]
	Serbia	56.6	2.91	2.5–33	[26]
Kidney	Germany	41.9	0.43		[41]
	Serbia	41.6	3.12		[27]
Liver	Germany	17	0.07		[22]
	Romania	75	0.16		[25]
	Serbia	46.6	2.88	up to 19.5	[27]
